# Digital religion in platform societies: authority, mediation, and social cohesion in algorithmic publics (2010–2025)

**DOI:** 10.3389/fsoc.2026.1802281

**Published:** 2026-04-17

**Authors:** Ismaael AlMazaedh, Khalaf M. Tahat, Mohannad Alkhalaileh, Dina N. Tahat

**Affiliations:** 1 Department of Arabic Language & Literature, United Arab Emirates University, Al Ain, United Arab Emirates; 2 Department of Arabic Language, The University of Jordan, Amman, Jordan; 3 Department of Media & Creative Industries, United Arab Emirates University, Al Ain, United Arab Emirates; 4 Department of Journalism and Digital Media, Faculty of Mass Communication, Yarmouk University, Irbid, Jordan; 5 College of Education, Humanities and Social Sciences, Al Ain University, Abu Dhabi, United Arab Emirates; 6 Department of Applied Sociology, College of Education, Al Ain University, Al Ain, United Arab Emirates

**Keywords:** algorithmic mediation, digital public sphere, digital religion, media governance, platform society, religious authority, social cohesion

## Abstract

This article critically synthesizes scholarship on digital religion and social cohesion published between 2010 and 2025. Drawing on a corpus of eighty-eight studies, we show that contemporary religious communication is increasingly governed by platform infrastructures, algorithmic visibility, and networked publics conditions that reshape religious authority, ritual practice, and community formation in ways that can both reinforce and fracture cohesion. Rather than treating platforms as neutral channels, the review conceptualizes digital religion as a layered socio-technical ecosystem whose outcomes hinge on the interaction between platform design, actor strategies, and audience participation. To integrate these literatures, the article advances the Platformed Cohesion Model (PCM), which specifies four interacting layers platform, actor, discourse, and cohesion through which digitally mediated religion produces variable effects on vertical cohesion (trust and legitimacy in institutions) and horizontal cohesion (solidarity and belonging among publics). The review concludes by identifying major gaps in cross-platform comparison, algorithm-centered inquiry, and audience-centered approaches, and proposes a research agenda for CMC scholarship that treats cohesion as an emergent property of platform-conditioned religious communication.

## 1 Introduction

Between 2010 and 2025, religion underwent a structural shift in how it is communicated, encountered, and legitimized. Authority increasingly moved from pulpits to platforms; ritual expanded from bounded sacred spaces to livestreams and modular clips; sermons and devotional guidance became optimized for visibility, engagement, and circulation. These transformations compelled scholars to reassess not only what counts as religious practice, but also how authority, identity, and community are negotiated under conditions of digitally mediated interaction (Campbell, 2012, 2017; Cheong, 2014a).

Early research often framed digital media as an auxiliary resource for religious institutions a set of tools used to extend reach and deliver content. Yet by the mid-2010s, scholarship increasingly emphasized that digital religion is not simply “religion online,” but an environment in which platform architectures shape the grammar of religious communication itself. Platforms such as YouTube, Instagram, X, and TikTok function as socio-technical actors: they organize visibility, discipline attention, incentivize performance, and assemble networked publics around religious narratives, symbols, and disputes (Campbell, 2012; Ess et al., 2012).

At the same time, social cohesion gained renewed urgency as societies confronted polarization, algorithmic segmentation, contested authority, generational divides, and intensified identity politics. Cohesion understood here as the relational infrastructure of trust, solidarity, belonging, and institutional legitimacy has become increasingly mediated by platform dynamics and online interaction. Religion is not peripheral to these tensions. Digitally circulated religious discourse can supply shared meaning, stabilize moral vocabularies, and mobilize solidarity; it can also amplify boundary-making, outrage cycles, and exclusionary identities when platform logics reward controversy and emotional intensity (Turkle, 2011; Papacharissi, 2011; Chan et al., 2006).

Against this backdrop, this article undertakes a structured critical narrative review of eighty-eight publications (2010–2025) to address a central question:

How does digital religion contribute to, or undermine, social cohesion in platform-based and algorithmically driven environments?

Rather than offering a descriptive inventory, the review maps the field's conceptual and empirical development, identifies key thematic clusters, surfaces contradictions, and proposes an integrative analytical framework that can guide CMC-oriented research on religion, platforms, and cohesion.

## 2 Review design and conceptual foundations

### 2.1 Review design and selection strategy

This article adopts a critical narrative review design to map the conceptual and empirical development of scholarship on digital religion and social cohesion between 2010 and 2025. It should be read as a critical narrative review rather than a systematic review: we follow transparent searching and screening procedures, but we prioritize conceptual synthesis and theory building across heterogeneous research designs (e.g., qualitative case studies, discourse analyses, ethnographies, surveys, and mixed-methods studies) rather than exhaustive coverage or meta-analytic aggregation.

The corpus comprises 88 published scholarly sources. To increase transparency and evaluability, we applied an iterative selection strategy: (1) identifying relevant publications through keyword searching and backward/forward citation tracking; (2) screening titles/abstracts for relevance to digital religion and cohesion-related dynamics (authority, ritual, community, trust, polarization, inclusion); and (3) extracting and coding each publication for platform focus, religious setting, methodological approach, and the type of cohesion claim advanced. Because evidence quality varies across methods and contexts, we treat methodological claims cautiously and foreground study limitations where they constrain inference. Table 1 summarizes the thematic clustering used for synthesis and provides representative works for each cluster.

**Table 1:** Thematic clusters used in the synthesis and representative works (illustrative).

Cluster	Core focus	Representative works (examples)
Authority and Leadership	How legitimacy is claimed, contested, and institutionalized online; mediated authority; platformed leadership.	Campbell, 2010; Cheong, 2014b; Hamdani, 2023; Zaid et al., 2022
Influencers and Performed Devotion	Religious micro-celebrities; branding; affective authenticity; devotion as performance in visibility economies.	Febrian, 2024; Buzid, 2020; Mannerfelt, 2022; Fuente Cobo et al., 2023
Ritual, Worship and Materiality	Online/offline ritual transformation; livestreamed worship; sensory and material mediation of the sacred.	Dein and Watts, 2023; Takala-Roszczenko, 2023; Taylor, 2022; Blackmer, 2024
Community, Belonging and Identity	Networked publics; identity performance; community-building and micro-public cohesion.	Papacharissi, 2011; Mattes et al., 2025; Hegazy and Abdelgalil, 2025; Urban and Orbe, 2010
Youth and Marginalized Publics	Generational dynamics; inclusion/exclusion; minority publics; migration and diasporic belonging.	Yust et al., 2010; Zaid et al., 2022; Lapis, 2025; Blackmer, 2024
Platform Infrastructures	Algorithms, affordances, monetization, moderation, and interface governance as structuring forces.	Postigo, 2011; Siuda, 2021; Müller, 2024; Abokhodair et al., 2020
Foundational Digital Religion Frameworks	Conceptual models of digital religion; mediation, practice theory, and the organization of the field.	Campbell, 2012, 2017; Ess et al., 2012; Lundby and Evolvi, 2022
Education, Ministry and Institutional Adaptation	Religious education, ministry, and organizational change in platform environments.	Drescher, 2012; Rankin, 2024; Tampubolon, 2023; Voelz, 2012

Clusters are analytical categories used for synthesis; many studies cut across clusters. Representative works are illustrative rather than exhaustive.

#### 2.1.1 Search strategy and sources

We consulted Scopus, Web of Science Core Collection, ATLA Religion Database, Communication and Mass Media Complete (EBSCOhost), JSTOR, and Google Scholar (last search: 15 January 2026). We used iterative keyword combinations that paired digital religion terms (e.g., “digital religion”, “religion online”, “online religion”, “networked religion”, “religion and social media”) with cohesion and identity terms (e.g., “social cohesion”, “social capital”, “belonging”, “trust”, “polarization”, “fragmentation”, “community”) and platform keywords (e.g., YouTube, Instagram, TikTok, Facebook, WhatsApp, Telegram, Discord, Twitter/X).

#### 2.1.2 Screening process

**Figure 1:**
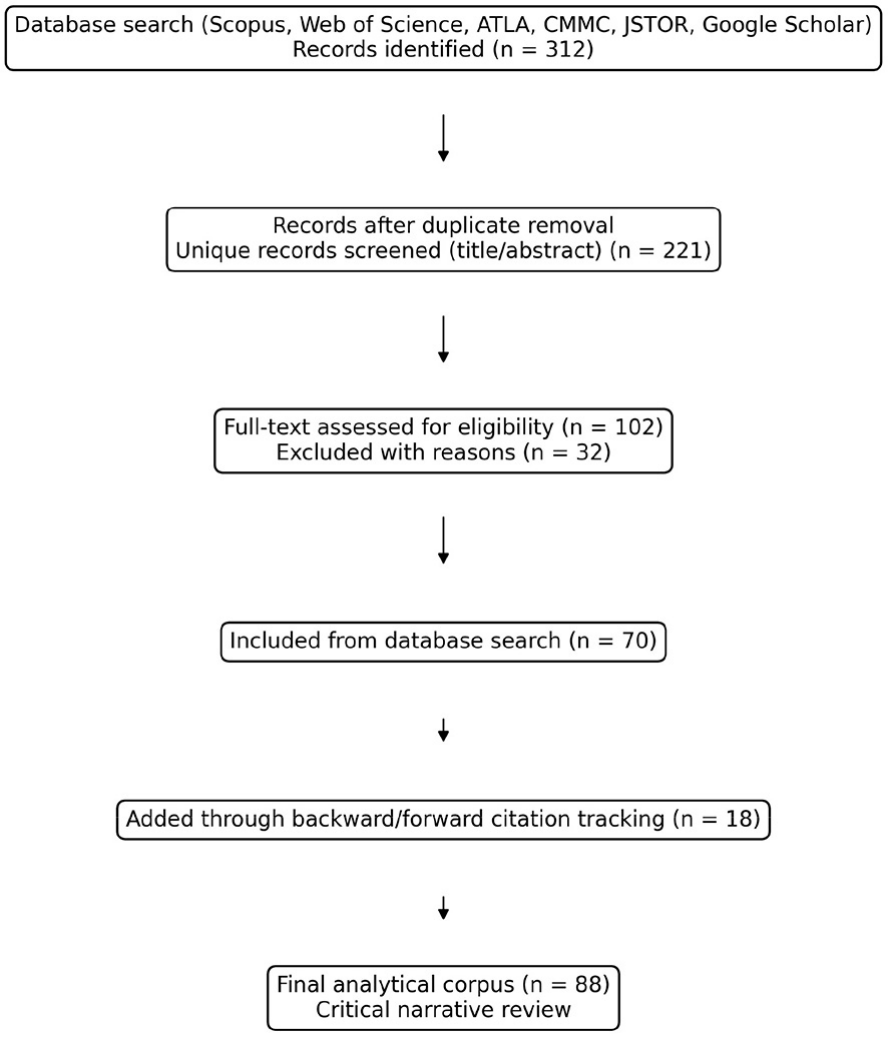
Literature search and screening process (critical narrative review).

The database search yielded 312 records. After deduplication (*n* = 221), titles and abstracts were screened for relevance to digital religion in platform societies (2010–2025). We then assessed 102 full texts against the inclusion criteria, resulting in 70 studies retained from the database search. Backward and forward citation tracking added 18 additional sources, bringing the final analytical corpus to 88 studies. Figure 1 provides a simplified flow diagram of this selection process. Because the article is a critical narrative review aimed at theory building, the procedure was transparency-oriented rather than a PRISMA-style systematic review.

To ensure analytical rigor, we explicitly grounded all synthesis in traceable in-text citations rather than generalized narrative summarization.

Inclusion criteria were: (a) publication between 2010 and 2025 (with a small number of pre-2010 works retained only for conceptual framing); (b) peer-reviewed journal articles, scholarly monographs, and published edited-book chapters; (c) explicit engagement with religion in relation to digital or social media platforms; and (d) an analytic link to at least one cohesion-relevant dimension (e.g., trust/legitimacy, solidarity/belonging, inclusion/exclusion, polarization/fragmentation). Exclusion criteria were: unpublished theses/dissertations and other gray literature, non-scholarly commentary (blogs/journalism), and works that do not substantively address platform environments or cohesion dynamics.

### 2.2 Platformed religious authority, influencers, and mediated legitimacy

Across traditions, digital platforms reconfigure religious authority by redistributing visibility and lowering barriers to entry for alternative voices. Institutional actors (e.g., churches, mosques, councils) can extend reach through official channels and livestreamed services, but they also operate in competitive attention environments where legitimacy is increasingly measured through engagement, affective resonance, and perceived authenticity (Campbell, 2010; Cheong, 2014b; Campbell, 2017).

Within this environment, influencer-style authority and “performed devotion” become consequential. Religious creators cultivate micro-publics through platform-native aesthetics (short video, visual piety, memes, and interactive Q&A), which can strengthen bonding and inclusion for youth publics, yet may also intensify boundary-making when identity is framed through oppositional discourse or when algorithmic incentives reward polarizing performances (Febrian, 2024; Buzid, 2020; Mannerfelt, 2022).

These dynamics highlight why competing frameworks such as “networked religion” and mediatization approaches remain important yet require updating for platform-specific infrastructures (algorithms, monetization, moderation) that structure contemporary religious communication. The Platformed Cohesion Model proposed later in this article builds on these foundations while foregrounding how platform architectures condition cohesion outcomes (Campbell, 2012; Hjarvard, 2011; van Dijck et al., 2018).

Positioning the Platformed Cohesion Model (PCM) against existing frameworks clarifies its added value. Hjarvard (2011) mediatization framework explains how media logics reshape religious institutions and practices, but it is not designed to model how distinct platform infrastructures translate into differentiated cohesion outcomes. van Dijck, Poell and de Waal (2018) platform society model foregrounds how platforms reorganize public values and governance, yet it remains largely sector-agnostic and offers limited conceptual leverage for mapping how religious authority, ritual, and discourse interact. Campbell and Garner (2016) networked theology (and related networked religion approaches) emphasize the relational negotiation of faith in digital networks, but they offer less direct guidance for connecting visibility regimes and discursive forms to vertical (institution–public) and horizontal (community–community) cohesion. PCM complements these approaches by (1) specifying a four-layer architecture (platform, actor, discourse, cohesion), (2) modeling recursive dynamics among these layers, and (3) making cohesion and fragmentation an explicit explanatory outcome enabling cross-platform comparison of why similar religious communication can generate trust and belonging in one setting and polarization or exclusion in another (Bucher, 2018; Gillespie, 2018; Campbell, 2017).

### 2.3 Ritual, materiality, and the digital sacred

Digital environments reshape ritual not by simply transferring worship from offline settings to online spaces, but by transforming how ritual time, ritual participation, and the sensory experience of the sacred are organized. The literature converges on three recurring transformations that are highly consequential for cohesion because they alter how shared meaning is experienced, repeated, and socially recognized (Dein and Watts, 2023; Takala-Roszczenko, 2023; Taylor, 2022).

First, ritual becomes a continuous symbolic flow rather than a bounded event. Livestreams, recorded sermons, short devotional clips, and algorithmically circulated prayers create a persistent stream that users can enter and exit at will. This shift changes ritual authority and ritual pacing, moving them away from scheduled collective time and toward platform-conditioned availability. In cohesion terms, the sacred becomes ambient: always potentially present, but not necessarily collectively synchronized (Taylor, 2022; Takala-Roszczenko, 2023).

Second, digital ritual becomes structurally participatory. Comments, reactions, emojis, reposting practices, and live chat interactions do not merely accompany ritual; they increasingly function as part of its performance and reception. The community becomes a co-producer of ritual visibility and affect. This matters because cohesion is not only about shared beliefs, but about shared interactional confirmation. When ritual is co-produced in real time, solidarity can intensify quickly. At the same time, the same interaction channels can become arenas of contestation, ridicule, or polarization, especially when visibility is reward-driven (Dein and Watts, 2023; Blackmer, 2024).

Third, the sensory profile of the sacred is reconfigured. Visual framing, editing, sound design, symbolic iconography, and platform aesthetics produce what the literature often frames as “visual piety,” where sacred meaning is experienced through media form as much as theological content. This sensory layering amplifies affective resonance and can broaden inclusion for audiences who may feel distant from traditional liturgical settings. Yet it can also compress complexity, favoring emotionally legible cues that platforms tend to reward (Grigore, 2025; McCorquodale and Sterten, 2010).

Together, these transformations show that digital ritual is not a neutral translation. It is a new ritual ecology, one that is materially shaped by platform affordances, interface cues, and engagement incentives, and therefore capable of generating either bridging cohesion or brittle, performative bonding.

### 2.4 Digital community and networked religious identity

In the platform age, religious community is increasingly decoupled from geographic proximity and formal institutional membership. Across the reviewed studies, community formation is consistently described as networked, interest-based, and organized through platform micro-publics rather than bounded congregational units. This does not mean offline communities disappear. It means that belonging is increasingly layered: users inhabit overlapping religious publics, each with its own norms, symbols, and trust cues (Papacharissi, 2011; Campbell, 2012; Zaid et al., 2022).

Digital religious identity, in this context, emerges as a performed and negotiated process shaped by recurring practices:

- participation in platform-specific micro-publics
- engagement with symbolic and aesthetic content
- interaction in thematic groups and networks
- personal remixing, circulation, and selective appropriation of religious meanings

This literature is particularly clear that digital community can provide high-value belonging for youth and marginalized publics. Mattes et al. (2025) and Zaid et al. (2022), for example, show that digital communities can function as alternative spaces of recognition, offering affiliation where traditional settings may impose exclusion or silence (Mattes et al., 2025; Zaid et al., 2022; Lapis, 2025).

However, this community logic has a cohesion tradeoff. Networked belonging can strengthen solidarity within micro-publics while weakening shared identity across broader publics, especially when algorithmic clustering encourages homophily and when moral boundaries are performed through visibility and public signaling. In other words, digital religious community can produce intense local cohesion and simultaneous societal fragmentation. That tension becomes central to the review's later analysis of horizontal cohesion (Turkle, 2011; Papacharissi, 2011; Hotait and Ali, 2024).

### 2.5 Platforms as active social and technical agents

A core insight across the corpus is that platforms are not passive channels for religious content. They operate as socio-technical agents that structure visibility, shape interaction, and indirectly define what counts as credible, meaningful, and shareable religion. Their infrastructures influence which voices rise, how publics gather, and how conflict escalates or de-escalates (Campbell, 2017; Siuda, 2021; Postigo, 2011).

To keep the analysis analytically disciplined, this review treats platforms as environments with distinct communicative grammars:

YouTube tends to support long-form religious communication, enabling extended sermons, lectures, and detailed explanations. This can reinforce institutional presence, while also enabling charismatic actors to bypass traditional gatekeeping and accumulate legitimacy via attention and search-based discovery (Fuente Cobo et al., 2023; Taylor and Evans, 2016).

Instagram centers aesthetic and symbolic communication. Religious identity becomes visually curated and stylized, often producing strong affective resonance. Yet the same aesthetic logic can simplify theological depth and shift authority toward branding and performance (Febrian, 2024; Zaid et al., 2022).

X amplifies brevity, virality, and emotionally charged expression. This format can facilitate rapid solidarity during crises, yet it also intensifies polarization by rewarding conflict-driven engagement (Cheong, 2010; Abokhodair et al., 2020).

TikTok privileges performance, rapid consumption, humor, and remix culture. Its visibility dynamics can empower youth-facing religious expression, while also pushing religious discourse toward spectacle and modularity (St. Lawrence, 2024; Hotait and Ali, 2024).

This platform-centered stance is not a stylistic preference. It is a theoretical necessity for a cohesion question. If social cohesion is partially produced through patterns of visibility, trust formation, and interactional norms, then platform architecture is not a background condition. It is part of the causal environment.

### 2.6 Toward an integrated conceptual lens (rebuilt)

Synthesizing the theoretical strands above, digital religion can be understood as a layered ecosystem in which:

Authority is negotiated through visibility, performativity, and audience interaction.

Ritual is reconfigured as fluid, participatory, and multimodal.

Community emerges through networked engagement and platform-conditioned micro-publics.

Platforms function as structuring agents that govern the circulation and reception of religious discourse.

This integrated lens is the conceptual bridge to the review's main analytical problem: how these platform-shaped transformations translate into cohesion outcomes. Put bluntly, the literature does not support a simple conclusion that digital religion is “good” or “bad” for cohesion. It supports a more rigorous claim: cohesion outcomes are produced by the interaction among platform infrastructures, actor strategies, discourse forms, and audience practices.

This is why the next section pivots from defining digital religion to operationalizing social cohesion in digital contexts, distinguishing vertical cohesion (trust and legitimacy between publics and institutions) from horizontal cohesion (solidarity and bonding within and across communities).

## 3 Digital religion and social cohesion

### 3.1 Understanding social cohesion in digital contexts

Social cohesion refers to the relational infrastructure that sustains collective life. Across sociological and communication research, it is commonly understood as a combination of trust, solidarity, shared norms, and perceived legitimacy that binds individuals to one another and to institutions. Within digitally mediated environments, these dimensions do not disappear, but they are reconfigured through platform-based interaction, algorithmic visibility, and networked participation (Chan et al., 2006; Schiefer and van der Noll, 2017).

For analytic clarity, this review distinguishes between two cohesion dimensions: vertical and horizontal (Chan et al., 2006; Schiefer and van der Noll, 2017). Vertical cohesion refers to the relationship between citizens and institutions (e.g., public trust, legitimacy, perceived fairness), and overlaps with work on linking social capital—ties that connect people to institutions and power holders (Szreter and Woolcock, 2004). Horizontal cohesion refers to the bonds among individuals and groups, encompassing shared identity, solidarity, and social trust within or across communities.

Beyond the vertical/horizontal axis, social capital scholarship highlights that cohesion operates through different kinds of ties. Putnam (2000) distinguishes between bonding social capital—dense, inward-looking ties that reinforce solidarity within relatively homogeneous groups—and bridging social capital—outward-looking ties that connect people across social, ideological, or religious difference. This distinction is directly relevant to digital religion, where platformed religious communication can strengthen bonding cohesion within devotional micro-publics while simultaneously weakening bridging cohesion by hardening group boundaries and reducing cross-cutting encounters (Putnam, 2000; Ellison et al., 2007; Williams, 2006).

In digitally mediated environments, bonding/bridging dynamics are not merely interpersonal; they are shaped by platform infrastructures that govern visibility, recommendation, and network formation. Accordingly, we conceptualize horizontal cohesion in PCM as comprising both bonding and bridging outcomes, while vertical cohesion captures trust in (and perceived legitimacy of) religious and civic institutions. This grounding helps specify why digital religion can generate solidarity and fragmentation at the same time—depending on whether platformed religious communication amplifies bonding at the expense of bridging (Putnam, 2007; Bucher, 2018; Gillespie, 2018).

Digital communication reshapes both dimensions simultaneously. Platforms extend institutional reach while exposing institutions to intensified scrutiny and contestation. They enable new forms of solidarity, but also foster segmentation, echo publics, and algorithmically amplified polarization. Importantly, cohesion in digital environments is not a stable condition. It fluctuates according to platform design, discursive framing, and audience practices (Papacharissi, 2011; Bucher, 2018; van Dijck et al., 2018).

Religion occupies a particularly sensitive position within these dynamics. Religious discourse often carries moral authority, symbolic density, and affective intensity. When such discourse circulates through platform infrastructures, it can stabilize collective meaning or intensify boundary drawing. Understanding digital religion therefore requires examining not only religious content, but the communicative environments through which cohesion is produced or eroded (Campbell, 2012; Evolvi, 2022; Cheong, 2014b).

### 3.2 Vertical cohesion: trust, legitimacy, and institutional mediation

Vertical cohesion is shaped by how digital religion restructures relationships between publics and religious institutions. The reviewed literature identifies three recurring patterns that capture this restructuring (Chan et al., 2006; Schiefer and van der Noll, 2017; Campbell, 2010; Cheong, 2014b).

First, digital platforms can reinforce institutional authority when religious organizations adapt effectively to platform logics. Official channels, livestreamed worship, and structured digital guidance systems can increase accessibility and visibility while maintaining doctrinal coherence. In moments of uncertainty or crisis, audiences often seek stable sources of guidance, and institutional digital presence can strengthen trust by offering continuity, reliability, and symbolic reassurance (Tari et al., 2021; Taylor, 2022; Rankin, 2024; Dein and Watts, 2023).

Second, platforms facilitate the emergence of parallel authority systems. Influencers and independent creators gain legitimacy through communicative skill, aesthetic resonance, perceived authenticity, and algorithmic reach rather than formal credentials. Research on Muslim millennials, Christian lifestyle influencers, and digitally mediated preaching demonstrates that these actors attract substantial followings and shape religious meaning outside institutional hierarchies. This diversification of authority expands interpretive options for audiences, but it also dilutes institutional monopoly over moral discourse (Zaid et al., 2022; Febrian, 2024; Hamdani, 2023; Mannerfelt, 2022).

Third, digital fragmentation can erode institutional dominance when religious organizations fail to align with platform cultures or audience expectations. Younger publics in particular often encounter contradictory religious messages across platforms, leading to selective trust and negotiated authority rather than deference. Institutional authority does not disappear in this environment, but it becomes contingent and continuously renegotiated (Hannan, 2023; Papacharissi, 2011; Turkle, 2011).

Taken together, these patterns show that vertical cohesion in digital religion is neither simply strengthened nor weakened. It is restructured into a hybrid condition where institutional legitimacy persists alongside influencer-driven authority and audience-based validation (Chan et al., 2006; Schiefer and van der Noll, 2017).

### 3.3 Horizontal cohesion: shared rituals, solidarity, and community formation

Horizontal cohesion concerns how digital religion shapes bonds among individuals and communities. The literature documents both strong integrative effects and significant risks of fragmentation (Chan et al., 2006; Schiefer and van der Noll, 2017).

One recurrent finding is that digital religion can generate solidarity through shared devotional practices. The circulation of prayers, sacred texts, and ritual symbols across platforms often produces moments of collective affect, particularly during crises such as pandemics, political upheaval, or communal loss. Livestreamed rituals and synchronous participation foster a sense of co-presence that transcends geographic boundaries (Dein and Watts, 2023; Taylor, 2022; Tari et al., 2021; Blackmer, 2024).

A second integrative effect concerns inclusion. Digital religious spaces provide alternative forms of belonging for youth, migrants, religious minorities, and individuals excluded from traditional congregational settings. These spaces allow flexible participation, anonymity, and identity experimentation. Studies repeatedly show that such environments can support resilience, recognition, and micro-level cohesion among marginalized publics (Mattes et al., 2025; Zaid et al., 2022; Lapis, 2025; Urban and Orbe, 2010).

At the same time, horizontal cohesion is vulnerable to fragmentation. Influencer-driven discourse may frame religious identity in oppositional terms, distinguishing authentic believers from outsiders or moral deviants. Algorithmic amplification intensifies this tendency by privileging emotionally charged or polarizing content. The result is often the consolidation of identity enclaves, sectarian narratives, and viral outrage cycles that weaken trust across communities (Abokhodair et al., 2020; Hotait and Ali, 2024; Papacharissi, 2011).

Digital religion therefore produces a paradox. It can strengthen solidarity within specific publics while undermining cohesion at broader societal levels. This paradox is central to understanding the ambivalent social role of religious communication in platform societies (Papacharissi, 2011; Abokhodair et al., 2020).

### 3.4 Ambivalent outcomes and context dependence

Across regions, platforms, and religious traditions, the reviewed studies converge on one conclusion: digital religion does not generate uniform cohesion outcomes. Its effects are context-dependent and shaped by the interaction of multiple factors (Campbell, 2017; Lundby and Evolvi, 2022).

Positive cohesion outcomes include expanded institutional reach, intensified communal solidarity, increased accessibility to religious resources, and strengthened support networks for diasporic and minority communities. These outcomes are most likely when institutional narratives, platform affordances, and audience practices align toward inclusion and constructive engagement (Blackmer, 2024; Zaid et al., 2022; Urban and Orbe, 2010).

Negative outcomes include polarization, erosion of institutional trust, misinformation, performative extremism, and fragmentation of previously cohesive communities. These effects are most pronounced when platform algorithms reward conflict, when influencers mobilize exclusionary identities, or when audiences are segmented into insulated micro-publics (Abokhodair et al., 2020; Bucher, 2018; Papacharissi, 2011).

Crucially, content alone does not determine outcomes. Cohesion emerges from how content is formatted, circulated, interpreted, and algorithmically amplified. Digital religion thus operates within a dual structure in which cohesion and fragmentation coexist as potential outcomes of the same communicative processes.

### 3.5 Linking digital religion to the structure of social cohesion

Synthesizing these findings allows several analytically grounded conclusions.

First, digital religion strengthens cohesion when platform infrastructures, actor strategies, and audience practices converge toward shared meaning and inclusive participation.

Second, it undermines cohesion when algorithmic visibility amplifies polarizing actors or when religious discourse is framed through exclusionary boundaries.

Third, cohesion is not static. It oscillates as platforms reorganize visibility, as actors adapt communicative strategies, and as audiences renegotiate trust and belonging.

Fourth, social cohesion in digital religion cannot be reduced to theological content or moral intention. It is a communicative outcome shaped by platform design, interactional norms, and socio-political context.

These insights provide the analytical foundation for the next section, which examines how specific platform logics structure religious communication and cohesion outcomes across YouTube, Instagram, X, and TikTok.

Platforms function as structuring agents that govern the circulation and reception of religious discourse.

Digital religion does not unfold in an abstract digital space. It evolves within platform-specific architectures that shape how religious meaning is produced, circulated, and interpreted. Across the reviewed literature, platforms emerge as active environments that structure visibility, regulate interaction, and privilege particular communicative forms. This section analyzes how platform logics shape religious authority, ritual expression, audience engagement, and ultimately social cohesion (van Dijck et al., 2018; Bucher, 2018).

To anchor this analysis, Table 2 provides a comparative overview of four platforms that dominate the literature on digital religion: YouTube, Instagram, X, and TikTok. The table should be read not as a typology of content, but as a map of communicative constraints and incentives that condition cohesion outcomes.

**Table 2:** Comparative overview of platform logics in digital religion (conceptual synthesis based on the reviewed literature).

Platform	Dominant mode	Key affordances	Authority effects	Ritual effects	Cohesion implications
YouTube	Long-form video	Searchability; scalability; monetization; playlists	Supports institutional legitimacy while enabling alternative long-form preachers	Extended teaching; livestreamed worship; structured ritual flows	Strengthens vertical cohesion; complicates horizontal cohesion through competing authorities
Instagram	Visual storytelling	Reels; stories; filters; aesthetic curation	Legitimacy through branding, symbolism, and affective appeal	Aestheticized devotion and symbolic piety	Strong micro-community bonding; weaker cross-group cohesion
X	Microblogging	Virality; hashtags; brevity; trending topics	Rhetorical authority and populist religious expression	Compressed ritual acts such as prayers, verses, and slogans	Rapid solidarity during crises; heightened polarization during conflict
TikTok	Short-form performance	Remix culture; music; editing tools; algorithmic velocity	Performative and youth-centered legitimacy	Modular ritual snippets and expressive devotion	Inclusion of youth publics; fragile and unstable cohesion

The focus on these platforms reflects their prominence in recent empirical research on digital religion and their central role in algorithmically mediated public discourse; in contrast, research on Facebook, Telegram, WhatsApp, and Discord remains comparatively underrepresented in the current peer-reviewed corpus, a limitation we highlight as a priority for future work.

## 4 Platform logics in digital religion

### 4.1 YouTube: institutional reach and alternative authority

YouTube is the most extensively examined platform in digital religion research. Its long form structure supports sustained religious communication, including sermons, theological lectures, and livestreamed rituals. For institutions, this format enables continuity with offline authority structures while expanding reach beyond physical congregations (Taylor and Evans, 2016; Fuente Cobo et al., 2023).

However, YouTube's open ecosystem also lowers entry barriers for independent actors. Charismatic preachers, religious educators, and influencers can accumulate legitimacy through narrative skill, emotional delivery, and search optimization rather than institutional affiliation. The literature repeatedly shows that audiences navigate between institutional and alternative authorities, often subscribing to both (Fuente Cobo et al., 2023; Taylor and Evans, 2016; Campbell, 2010).

From a cohesion perspective, YouTube strengthens vertical cohesion by preserving recognizable authority formats. At the same time, it fragments horizontal cohesion by multiplying interpretive centers and encouraging selective allegiance. Authority becomes layered rather than singular, and communal alignment becomes more difficult to sustain at scale.

### 4.2 Instagram: visual piety and symbolic identity

Instagram reshapes religious communication through its emphasis on visual aesthetics and symbolic condensation. Religious authority on this platform is frequently constructed through curated imagery, motivational captions, and lifestyle oriented self-presentation. This produces what the literature describes as visual piety, where devotion is experienced through aesthetic coherence and affective resonance (Febrian, 2024; Zaid et al., 2022).

Instagram facilitates strong bonding within micro-communities organized around shared taste, tone, and symbolic repertoires. These communities often generate high levels of affective cohesion and perceived intimacy.

At the same time, the platform's aesthetic logic can reduce theological complexity and privilege personal branding over collective meaning. Authority becomes individualized, and cohesion tends to remain localized rather than broadly integrative. The result is intense micro-level cohesion accompanied by limited capacity for cross-community integration.

### 4.3 X: crisis rituals, populist religion, and fragmentation

X amplifies religious communication through brevity, immediacy, and algorithmic virality. Religious discourse on this platform often intensifies during moments of crisis, when prayers, supplications, and sacred texts circulate rapidly. These moments generate ambient solidarity and short-lived horizontal cohesion (Cheong, 2010; Abokhodair et al., 2020).

Yet the same features that enable rapid bonding also intensify conflict. The platform's reward structure privileges emotionally charged, controversial, or oppositional content. Religious discourse easily becomes entangled with political polarization, identity signaling, and moral boundary drawing.

The literature consistently identifies X as a platform where cohesion oscillates sharply. Moments of unity are followed by cycles of fragmentation, outrage, and symbolic conflict. Cohesion here is volatile, situational, and easily disrupted (Cheong, 2010; Abokhodair et al., 2020; Papacharissi, 2011).

### 4.4 TikTok: performed devotion and algorithmic identity

TikTok represents the most recent and rapidly evolving platform in digital religion research. Its design centers on performance, speed, remixing, and affective intensity. Religious content on TikTok often blends devotion with humor, music, and dramatic expression (St. Lawrence, 2024; Hotait and Ali, 2024).

Authority on TikTok is highly performative. Legitimacy emerges through emotional resonance, relatability, and algorithmic reach rather than doctrinal expertise. Ritual becomes modular and replicable, circulating as short expressive units rather than structured liturgical sequences.

From a cohesion perspective, TikTok attracts youth publics and facilitates inclusion for those alienated from institutional religion. However, the cohesion it produces is fragile. Trends change rapidly, identities are expressed rather than stabilized, and algorithmic clustering can intensify polarization across subcultures (St. Lawrence, 2024; Hotait and Ali, 2024).

### 4.5 Cross platform patterns of cohesion

To synthesize these platform specific dynamics, Table 3 summarizes how digital religion shapes cohesion across the four platforms.

**Table 3:** Platform effects on dimensions of social cohesion (conceptual synthesis; not a quantitative scale).

Platform	Vertical cohesion	Horizontal cohesion	Fragmentation	Inclusive belonging
YouTube	High support through institutional channels	Moderate through shared rituals	Medium due to competing authorities	High
Instagram	Low to moderate due to influencer dominance	High within micro-communities	Medium due to identity branding	Moderate
X	Moderate during crises	High during crises; low during conflicts	High due to polarization	High in crisis contexts
TikTok	Low due to youth-driven performativity	Moderate among youth; unstable overall	High due to algorithmic enclaves	High for youth subcultures

### 4.6 Platforms as architectures of cohesion

Across the literature, platforms function less as channels and more as architectures of cohesion. They determine which religious voices gain visibility, how rituals are experienced, and how publics assemble and fragment (van Dijck et al., 2018; Bucher, 2018; Gillespie, 2018).

Three analytical insights emerge. First, platform design shapes the symbolic form of religious discourse. Second, algorithmic visibility governs authority formation. Third, audience practices interact with platform logics to produce variable cohesion outcomes (van Dijck et al., 2018; Bucher, 2018).

These insights prepare the ground for the next section, which introduces the Platformed Cohesion Model as a unified framework synthesizing 15 years of scholarship into a coherent explanatory structure.

## 5 The Platformed Cohesion Model (PCM) rebuilt as a communication framework

The expansion of digital religion between 2010 and 2025 has produced a dense but fragmented body of scholarship. While individual studies illuminate specific platforms, actors, or practices, the field lacks a unifying framework capable of explaining how these elements interact to produce cohesion or fragmentation across contexts. The Platformed Cohesion Model (PCM) is proposed to address this gap.

PCM conceptualizes digital religion as a layered communicative ecosystem in which platform infrastructures, religious actors, symbolic discourse, and cohesion outcomes are dynamically interconnected. Rather than treating cohesion as an abstract social effect, the model situates it as an emergent outcome of mediated interaction shaped by platform logic and audience engagement.

**Figure 2:**
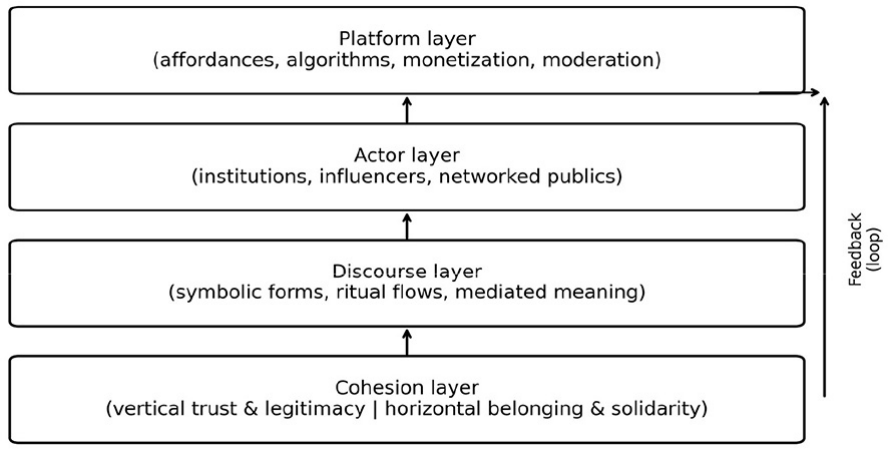
The Platformed Cohesion Model (PCM): a four-layer framework linking platform infrastructures, actors, discourse, and social cohesion outcomes.

The model consists of four analytically distinct but interdependent layers: the platform layer, the actor layer, the discourse layer, and the cohesion layer. Figure 2 visualizes these layers and their recursive feedback loops.

### 5.1 The platform layer: infrastructure, affordances, and algorithmic visibility

The platform layer constitutes the structural foundation of the model. Platforms define the communicative environment in which religious interaction takes place by regulating visibility, interaction, and circulation.

Across the literature, four infrastructural elements repeatedly appear as decisive. Algorithmic visibility determines which religious voices become prominent and which remain marginal. Affordances such as video length, editing tools, and interactivity shape the expressive possibilities available to religious actors. Economic incentives such as monetization and influencer economies affect content production strategies. Interface design influences how audiences encounter, interpret, and evaluate religious messages (van Dijck et al., 2018; Bucher, 2018; Gillespie, 2018).

At this layer, platforms are not neutral intermediaries. They function as governing environments that privilege specific communicative forms. Long form exposition, aesthetic symbolism, rhetorical brevity, or performative intensity are not merely stylistic choices. They are structural outcomes of platform design.

The platform layer therefore establishes the conditions under which authority can be claimed, rituals can be enacted, and communities can emerge.

### 5.2 The actor layer: institutions, influencers, and networked publics

The second layer focuses on the actors who operate within platform infrastructures. The literature consistently identifies three dominant actor categories (Campbell, 2017; Cheong, 2014b; Papacharissi, 2011).

Institutional actors include churches, mosques, religious councils, and official ministries that adapt established authority structures to digital environments. Their primary objective is to preserve legitimacy and sustain vertical cohesion by translating offline authority into online formats (Drescher, 2012; Tampubolon, 2023; Rankin, 2024; Taylor, 2022).

Influencers and independent creators represent a second category. These actors gain legitimacy through visibility, perceived authenticity, and engagement metrics rather than institutional credentials. Their authority is performative and audience dependent. They often blend devotion with lifestyle content, emotional narrative, or entertainment formats (Febrian, 2024; Buzid, 2020; Fuente Cobo et al., 2023; Mannerfelt, 2022).

Networked publics form the third category. Audiences are not passive recipients of religious messages. They co-produce meaning through comments, remixing, sharing, and selective trust. Their engagement feeds back into algorithmic systems, shaping future visibility and authority hierarchies (Papacharissi, 2011; Campbell, 2012; Abokhodair et al., 2020).

The actor layer highlights a fundamental shift documented across the literature. Authority in digital religion emerges through communicative performance and audience recognition rather than hierarchical position (Campbell, 2010; Cheong, 2014b; Campbell, 2017).

### 5.3 The discourse layer: symbolic forms, ritual flows, and mediated meaning

The third layer addresses the symbolic and communicative forms that circulate across platforms. Digital religious discourse is multimodal, performative, and highly responsive to platform constraints (Taylor, 2022; Dein and Watts, 2023; Febrian, 2024).

The reviewed studies identify recurring discursive forms including audio visual sermons, stylized devotional imagery, short ritual videos, mediated sacred texts, influencer narratives, meme-based theology, and crisis driven prayer flows. These forms are not interchangeable. Each carries distinct implications for how the sacred is experienced and how communal boundaries are drawn (Taylor and Evans, 2016; Febrian, 2024; St. Lawrence, 2024; Buzid, 2020).

At this layer, ritual is no longer a bounded event but a continuous communicative flow. Meaning becomes portable, remixable, and context sensitive. The sacred is experienced through affective intensity, aesthetic coherence, and participatory interaction rather than spatial enclosure (Taylor, 2022; Takala-Roszczenko, 2023; Dein and Watts, 2023).

This transformation reshapes how individuals construct religious identity and how communities negotiate inclusion and exclusion (Mattes et al., 2025; Zaid et al., 2022; Lapis, 2025).

### 5.4 The cohesion layer: vertical and horizontal outcomes

The cohesion layer captures the collective outcomes of platformed religious communication. In PCM, cohesion refers to the presence (or erosion) of trust, solidarity, and shared identity among individuals and groups. The model distinguishes between vertical cohesion (cohesion between citizens and institutions) and horizontal cohesion (cohesion among citizens and communities), while also recognizing that horizontal cohesion may take bonding or bridging forms (Chan et al., 2006; Schiefer and van der Noll, 2017; Putnam, 2000).

Vertical cohesion refers to trust, legitimacy, and alignment between institutions and publics. It strengthens when institutional actors effectively leverage platform affordances and align their discourse with audience expectations. It weakens when influencer authority eclipses institutional credibility or when contradictory religious narratives proliferate unchecked (Chan et al., 2006; Schiefer and van der Noll, 2017; Cheong, 2014b; Hamdani, 2023).

Horizontal cohesion refers to solidarity and shared identity among individuals and communities. In the context of digital religion, it includes bonding cohesion (strengthening in-group ties within smaller publics) and bridging cohesion (cross-cutting ties across groups and traditions). Platform dynamics can reinforce horizontal cohesion through supportive networks and collective ritual participation, but may also weaken it through polarization, fragmentation, and conflict escalation (Putnam, 2000; Chan et al., 2006; Papacharissi, 2011).

In PCM, cohesion is not a stable outcome. It is dynamic, oscillating as platform logic, actor strategies, and discourse forms interact over time (Chan et al., 2006; Schiefer and van der Noll, 2017).

### 5.5 Recursive interaction among the four layers

A central contribution of PCM lies in its emphasis on recursion. The four layers do not operate sequentially but interact continuously (Bucher, 2018; van Dijck et al., 2018).

Platform infrastructures shape actor behavior. Actor strategies shape discourse. Discourse influences audience interpretation and engagement. Engagement feeds back into platform algorithms, reshaping visibility and authority (Bucher, 2018; van Dijck et al., 2018; Gillespie, 2018).

This recursive dynamic explains why similar religious content can produce divergent cohesion outcomes across platforms and contexts. It also accounts for rapid shifts in authority and community alignment observed in digital religious environments (Campbell, 2017; Bucher, 2018; van Dijck et al., 2018).

For example, algorithmic promotion of high emotion content incentivizes performative devotion. Performative devotion attracts youth publics. Youth publics form expressive micro-communities. These communities reinforce algorithmic clustering, intensifying fragmentation at the societal level (Bucher, 2018; St. Lawrence, 2024).

PCM thus captures digital religion as an evolving communicative system rather than a collection of isolated practices (Campbell, 2017; Siuda, 2021).

### 5.6 Analytical value of the PCM model

The Platformed Cohesion Model offers three core contributions.

First, it provides a unified analytical framework that integrates platform studies, communication theory, and religion research. Second, it offers explanatory power by clarifying how infrastructural conditions and communicative practices jointly shape cohesion outcomes. Third, it establishes a foundation for future research designs, including comparative platform analysis, audience centered ethnography, and algorithmic investigation.

By shifting attention from content alone to the layered interaction of platforms, actors, discourse, and cohesion, PCM repositions digital religion as a central case for understanding communication in platform societies.

## 6 Research gaps and future directions reframed as a field agenda

Despite rapid growth between 2010 and 2025, scholarship on digital religion and cohesion remains conceptually dispersed and methodologically uneven. Individual studies offer rich insight into platforms, actors, and practices, yet cumulative theory building is still limited. Building on PCM, we highlight the most consequential gaps that constrain explanation and outline a focused research agenda (Campbell, 2017; Lundby and Evolvi, 2022).

### 6.1 Cross platform blindness and the fragmentation of evidence

A dominant limitation across the reviewed literature is platform isolation. Many studies focus on a single platform (often YouTube or Instagram) and under-specify cross-platform circulation and audience migration processes that can reshape authority formation and cohesion dynamics.

As a result, cohesion outcomes are sometimes attributed to content alone rather than to infrastructural translation (e.g., how formats, affordances, and ranking systems change meanings as discourse moves between platforms).

Future research should prioritize explicitly cross-platform designs (comparative and trace-based) that follow religious messages, actors, and publics across platform ecosystems to test whether cohesion effects travel with content or emerge from platform-specific infrastructures.

### 6.2 The algorithmic black box and the absence of infrastructure analysis

Although many studies acknowledge algorithms, few empirically examine recommendation, moderation, and monetization systems. This leaves visibility regimes and authority formation under-specified and weakens causal claims about cohesion and fragmentation (Bucher, 2018; Gillespie, 2018; van Dijck et al., 2018).

Without infrastructure-level analysis, it remains difficult to explain why particular religious voices dominate, how harms (misinformation/sectarianism) are governed, and why similar content produces divergent outcomes across contexts.

Future research should integrate platform-studies and computational approaches (e.g., pathway audits, interface ethnography, and policy analysis) to make governance and visibility empirically observable and comparable across platforms.

### 6.3 Audience marginalization in digital religion scholarship

Much of the literature privileges institutions and influencers while treating audiences as aggregate metrics. Yet audiences interpret, contest, and remix religious content; their participation is central to trust, belonging, and polarization dynamics (Papacharissi, 2011; Campbell, 2012).

Audience practices commenting, sharing, remixing, and selective trust often determine whether digital religion fosters bridging cohesion, brittle bonding, or fragmentation.

Future studies should adopt audience-centered designs (digital ethnography, comment/repost analysis, and longitudinal reception tracking) to show how cohesion is co-produced through everyday interaction rather than exposure alone.

### 6.4 Temporal compression and the absence of longitudinal insight

Evidence is also temporally compressed. Many studies are crisis snapshots (e.g., COVID-19), which limits insight into the durability of online rituals, the lifecycle of influencers, and longer-term institutional adaptation (Tari et al., 2021; Taylor, 2022).

Without longitudinal analysis, it remains unclear whether digital religious practices stabilize, institutionalize, or dissipate over time, and how cohesion effects evolve as platforms change.

Longitudinal and panel designs are needed to distinguish episodic cohesion from sustained communal transformation and to track how platform updates reshape religious publics.

### 6.5 Geographic imbalance and the limits of Western theory

Despite growing diversity, scholarship remains heavily weighted toward Western/Christian contexts; Global South research is expanding but is still underrepresented in theory building.

This imbalance risks universalizing Western assumptions and overlooking alternative configurations of authority, public religion, and state relations; non-Western cases often reveal hybrid authority structures and distinct cohesion dynamics.

Future research should treat non-Western contexts as theory-generating and use comparative designs to examine how platform governance intersects with local religious-political arrangements.

### 6.6 Gender, youth, and intersectional blind spots

Gender and youth are central to platform religion (performance, aesthetics, influencer economies) yet are still peripheral in much field-level theorizing (Febrian, 2024; Mattes et al., 2025).

Because platformed religion is deeply shaped by gendered performance and youth-centered aesthetics, ignoring these dimensions limits explanatory depth for both inclusion and fragmentation outcomes.

Intersectional frameworks are needed to explain how cohesion outcomes vary by gender, age, class, and minority status across platform environments.

### 6.7 Theoretical fragmentation and the need for integrative models

Digital religion research also suffers from theoretical fragmentation: studies draw on multiple disciplines without consistent integration, limiting cumulative explanation (Campbell, 2017; Lundby and Evolvi, 2022).

PCM responds to this challenge by integrating platform infrastructures, actors, discourse forms, and cohesion outcomes into a single explanatory frame, but the model requires empirical testing and refinement.

Model-driven research can operationalize PCM layers and test their interactions empirically through comparative platform analysis and methodological triangulation.

### 6.8 Toward a mature research program

Taken together, these gaps define a research agenda rather than a deficit. Advancing the field requires integrated and comparative designs, infrastructure-aware analysis, audience-centered methods, and greater geographical and intersectional breadth.

Pursuing these priorities can move digital religion scholarship beyond descriptive mapping toward cumulative explanation and theory building.

## 7 Conclusion repositioned for editorial closure

Between 2010 and 2025, digital religion shifted from a marginal research interest to a central analytical lens for understanding how meaning, authority, and belonging are negotiated in platform mediated societies. This review synthesized 88 studies across platforms, regions, and religious traditions to map how religious communication intersects with social cohesion under conditions of algorithmic mediation and networked visibility.

The analysis demonstrates that digital religion cannot be classified as inherently cohesive or inherently divisive. Instead, its social effects are contingent upon the interaction between platform architectures, actor strategies, symbolic discourse, and audience practices. Platforms shape the conditions of visibility. Actors negotiate legitimacy through performance and engagement. Discourse circulates as multimodal ritual and symbolic flow. Cohesion emerges as a dynamic and unstable outcome of these layered interactions.

The Platformed Cohesion Model provides a unifying framework capable of integrating these dynamics. By conceptualizing digital religion as a multi-layer communicative ecosystem, the model clarifies why similar religious content produces divergent cohesion outcomes across platforms and contexts. It also explains how authority becomes distributed, rituals become portable, and communities become simultaneously expansive and fragmented.

The reviewed literature shows that digital religion can strengthen vertical cohesion by extending institutional reach, enhancing accessibility, and providing stable sources of guidance during periods of uncertainty. It can also foster horizontal cohesion through shared rituals, affective solidarity, and inclusive digital communities that support marginalized publics. At the same time, digital religion can intensify fragmentation when platform logics amplify polarizing discourse, reward performative extremism, or cluster audiences into algorithmic enclaves (Tari et al., 2021; Dein and Watts, 2023; Zaid et al., 2022; Abokhodair et al., 2020; Bucher, 2018).

These findings challenge linear assumptions about the social role of religion in digital environments. They suggest that cohesion is not a fixed property of religious communication, but an emergent condition shaped by infrastructural design and communicative practice. Digital religion thus operates as a revealing case for broader transformations in public communication, authority formation, and collective identity in platform societies.

Ultimately, the study of digital religion offers more than insight into religious practice. It provides a window into how cohesion and fragmentation are produced in an era where platforms mediate the sacred and the social alike. Understanding these dynamics is essential for advancing communication theory and for grasping how trust, belonging, and authority are reconfigured in the digital age.

## 8 Implications for scholarship and policy recalibrated

The findings of this review extend beyond descriptive synthesis and carry implications for theory building, institutional practice, and public policy. As digital religion becomes embedded within platform infrastructures, its role in shaping cohesion, authority, and belonging acquires broader significance for communication scholarship and governance.

### 8.1 Implications for communication scholarship

First, the review underscores the need for theoretical consolidation in digital religion research. The Platformed Cohesion Model demonstrates that religious communication cannot be adequately explained through platform specific or content centered approaches alone. Scholars are encouraged to adopt layered frameworks that integrate platform infrastructures, actor strategies, symbolic discourse, and social outcomes.

Second, the findings call for methodological pluralism. Advancing the field requires research designs that combine digital ethnography, discourse analysis, audience studies, and computational methods. Algorithmic visibility, engagement metrics, and platform governance must be treated as empirical variables rather than background conditions.

Third, the review highlights the importance of global and intersectional perspectives. Digital religion research must move beyond Western centric assumptions and incorporate diverse religious traditions, political contexts, and cultural configurations. Gender, youth, class, and minority identities should be central analytical categories rather than peripheral considerations.

Finally, the PCM model offers a foundation for cumulative research. Scholars can operationalize its layers to test hypotheses across platforms, regions, and time frames. In doing so, digital religion can serve as a generative case for broader theories of computer mediated communication.

### 8.2 Implications for religious institutions and civil society

For religious institutions, the findings suggest that authority in digital environments is increasingly negotiated rather than inherited. Maintaining trust and legitimacy requires communicative competence, platform literacy, and responsiveness to audience expectations. Institutions that adapt ritual formats, embrace interactivity, and cultivate inclusive digital spaces are more likely to sustain cohesion.

Civil society organizations working at the intersection of religion and community building can leverage digital platforms to foster dialogue, solidarity, and resilience. However, such initiatives must remain attentive to platform logics that may inadvertently amplify exclusionary or polarizing discourse.

### 8.3 Implications for policy and platform governance

At the policy level, the review points to the growing importance of platform governance in shaping religious expression and social cohesion. Regulators and platform providers must balance religious freedom with the mitigation of harm, including misinformation, hate speech, and sectarian incitement (Gillespie, 2018; Bucher, 2018).

Policies that promote transparency in content moderation, accountability in algorithmic design, and protection for minority religious voices can contribute to more cohesive digital publics. Additionally, public investment in digital literacy programs can empower users to critically engage religious content and navigate platform dynamics more effectively.

### 8.4 Toward cohesion in platform mediated societies

Digital religion illustrates how platforms have become central infrastructures of public life. The implications of the Platformed Cohesion Model therefore extend beyond religious studies. They speak to fundamental questions about how trust, belonging, and authority are constructed in societies where communication is increasingly mediated by algorithmic systems.
